# Recent advances in vascularized tumor-on-a-chip

**DOI:** 10.3389/fonc.2023.1150332

**Published:** 2023-03-30

**Authors:** Christina Bao Xian Huang, Ting-Yuan Tu

**Affiliations:** ^1^ Department of Biomedical Engineering, National Cheng Kung University, Tainan, Taiwan; ^2^ Medical Device Innovation Center, National Cheng Kung University, Tainan, Taiwan; ^3^ International Center for Wound Repair and Regeneration, National Cheng Kung University, Tainan, Taiwan

**Keywords:** organ-on-a-chip, tumor-on-a-chip, vascularized tumor-on-a-chip, microfluidics, vasculogenesis

## Abstract

The vasculature plays a critical role in cancer progression and metastasis, representing a pivotal aspect in the creation of cancer models. In recent years, the emergence of organ-on-a-chip technology has proven to be a robust tool, capable of replicating *in vivo* conditions with exceptional spatiotemporal resolution, making it a significant asset in cancer research. This review delves into the latest developments in 3D microfluidic vascularized tumor models and their applications *in vitro*, focusing on heterotypic cellular interactions, the mechanisms of metastasis, and therapeutic screening. Additionally, the review examines the benefits and drawbacks of these models, as well as the future prospects for their advancement.

## 1 Introduction

Despite substantial efforts and advancements in cancer research, many of the mechanisms that drive cancer progression remain to be elucidated. There is currently no model that can perfectly recapitulate all of the components of the tumor microenvironment (TME). The most widely used tools for cancer research are currently animal models and 2D cell cultures. Although 2D cell culture offers simplicity and high throughput, it does not recapitulate the complex interaction between cells in the TME. Although animal models, which are the current gold standard, can provide a complex TME, it is not uncommon for the results to have a poor correlation to biological response in humans, leading to a high failure rate of drugs in clinical trials (1). This phenomenon might be due to various factors, such as species differences, which are becoming more important as an increasing number of biologics and cell-based therapies are being developed, and the use of immunocompromised models, which are different from cancer patients who still have a functional immune system. Furthermore, animal experiments usually have a longer testing period, are costly, and are subject to tighter regulations in terms of subject numbers due to ethical concerns.

It is well established now that the tumor has a complex and dynamic microenvironment comprising cancer cells, stroma cells, and other abiotic components. Vasculature is one of the key components affecting tumor progression and treatment response (2, 3). Over the past decade, microfluidic models have emerged to bridge the gap between traditional *in vitro* models and *in vivo* models. It is a robust tool that better mimics human physiological and pathological conditions *in vitro*, including cancer (4, 5). The modular nature of microfluidic systems offers the ease of incorporating different cell types and controlling biochemical and biophysical factors such as concentration gradients and flow. This allows researchers to delineate the role of different players and to elucidate emerging behaviors more conveniently. In addition, it offers higher throughput, spatiotemporal control, and resolution compared to traditional *in vivo* models.

In addition to delivering oxygen, nutrients, and drugs, as well as removing metabolic wastes, the vasculature also serves as an important route for tumor metastasis, which accounts for approximately 90% of cancer-associated deaths (6). After invading the surrounding tissue, cancer cells reach the vasculature and intravasate, becoming circulating tumor cells (CTCs). CTCs circulate throughout the body either as single cells or as clusters and may associate with other cells circulating in the bloodstream. Upon arrest, they may extravasate from the vessel and potentially form a secondary tumor. The design of vascularized tumor-on-a-chip models can be customized to elucidate the mechanisms of the various steps of cancer metastasis.

With the abovementioned advantages, microfluidic vascularized tumor-on-a-chip models also hold great potential for therapeutic screening applications. The presence of vasculature has been shown to improve drug or engineered immune cell trafficking to the tumor (3). Moreover, the minute working volume of microfluidic devices is excellent for handling valuable samples. Vascularized tumor-on-a-chip can be designed to match the 96- or 384-well format compatible with high-throughput machinery, making it an attractive tool for drug screening. It is also convenient for assessing the dynamic TME continuously or periodically.

In this review, we first introduce the state-of-the-art strategies for vascularizing tumors-on-a-chip and then elaborate on the recent advances using vascularized tumors-on-a-chip for different applications including heterotypic cellular interactions in the TME, unveiling the mechanisms of the metastatic cascade, and therapeutic screening. Finally, we conclude this review by discussing the advantages, limitations, and future directions for vascularized tumor-on-a-chip development.

## 2 Vascularization strategies

To establish vascularized tumor models in microfluidic devices, vasculature can be generated separately or together with the tumor (single cells or spheroids). To generate the vasculature itself, three different methods can be used—endothelial cell (EC) lining, vasculogenesis, and angiogenesis. EC lining usually generates vasculature at the mesoscale (approximately a few hundred micrometers in diameter), while vasculogenesis and angiogenesis produce a narrower vessel lumen, which better resembles capillaries *in vivo*.

### 2.1 Endothelial cell lining

**Figure 1:**
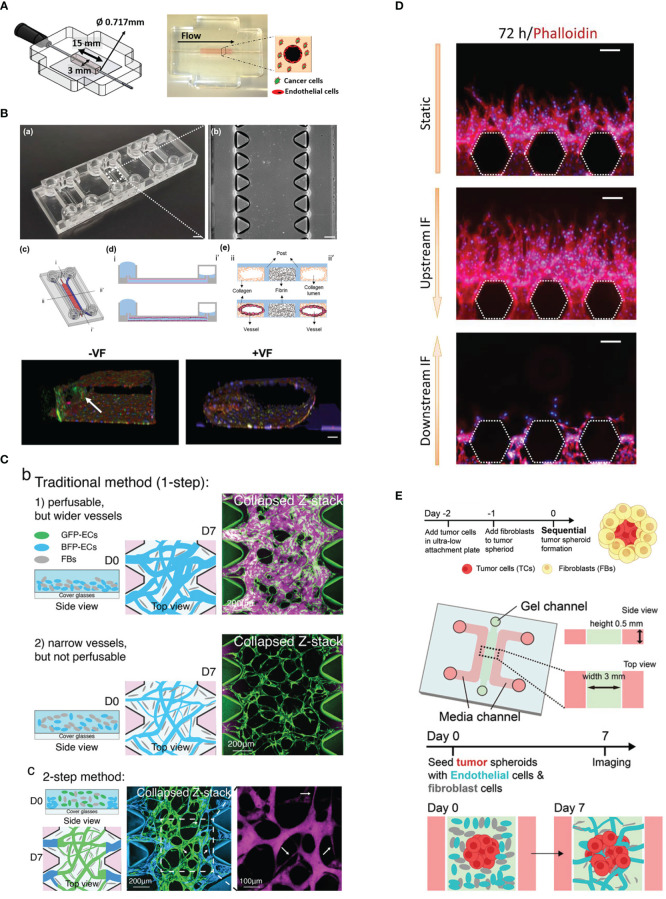
Vascularization strategies. **(A)** Endothelial cell lining on a gel channel pre-patterned using needle. Adapted with permission. Copyright 2020 Wiley Periodicals LLC. **(B)** The viscous fingerprinting method is used to line the microfluidic channels, ensuring a more even endothelial cell coverage. Adapted under the terms of the Creative Commons Attribution License (CC BY). Copyright 2022 Tu et al. **(C)** The two-step vasculogenesis method allows the formation of a narrower vessel in the middle and good opening to the lateral channels. The top part and middle part show the vessel formed under the traditional method with high and low density of EC, respectively. Adapted with permission. Copyright 2022 Wan, Zhong, et al. Small Methods published by Wiley‐VCH GmbH. **(D)** Sprouting angiogenesis is enhanced by interstitial flow from upstream. Adapted under the terms of the Creative Commons Attribution License (CC BY). Copyright 2022 Liu et al. **(E)** The sequential seeding of fibroblast on tumor spheroid enhances tumor vascularization. The vascularized tumor model is used for evaluating CAR T performance. Adapted with permission. Copyright 2022 Wan, Floryan, et al. Advanced Healthcare Materials published by Wiley‐VCH GmbH.

Usually, ECs can be lined on the outer side of a gel channel, on a membrane in a vertically stacked model (7), or on the inner side of a prepatterned lumen (**Figure 1A**). EC seeding density from 1 million cells/ml (8) to 20 million cells/ml (9) has been reported, usually ranging about a few million cells per ml, possibly due to the differences in geometry of the surface to be lined and other conditions. The vessel will be ready to use for experiments as soon as 24 h postseeding, depending on the seeding density, and can be sustained for a few more days before the ECs overgrow. A past application showed that the vessel had been maintained for up to 16 days to investigate the angiogenic potential of inflammatory breast cancer (IBC) cells (10). Another advantage of this method is the more consistent and reproducible geometry. However, the vessel diameter of a few hundred micrometers is much larger than that of the capillaries *in vivo*. It is also convenient that several different types of ECs can be used for this method.

In the lumen prepatterning method, a microneedle or rod of various diameters can be inserted into the pregel and is carefully removed after the gel has polymerized. Alternatively, lumen formation in microfluidic channels can be achieved with the viscous finger patterning method (11, 12), where the gel is first injected into the microfluidic channel and subsequently displaced with less viscous fluid, creating a hollow lumen upon gel polymerization (**Figure 1B**). This step is performed to prevent heterogeneity in the EC monolayer between the part attached to the gel interface and the microfluidic post if directly seeded.

### 2.2 Self-assembling methods

To obtain a more natural vessel morphology, self-assembling methods can be utilized. These methods usually achieve vascularization around the tumor and even into the tumor interior.

#### 2.2.1 Vasculogenesis

Vasculogenesis is the process of *de novo* formation of vessels in the presence of endothelial (progenitor) cells at high density. For *in vitro* vasculature formation, generally seeded at a density between 5 to 10 million cells/mL, together with supporting cells. Fibroblasts are the most widely used cell stromal cells, although other cell types have also been used depending on the microenvironment to be mimicked (13–18). The main reason for incorporating stromal cells is to support the formed microvasculature, which will otherwise regress after initial formation in the EC monoculture (19).

In vasculogenesis, using a high EC density tends to result in more opening to the lateral channel. Using high EC density, however, will result in larger vessel diameter, which is different from the small capillaries (under 10 µm) *in vivo*. Recently, a two-step seeding method (20) was established to address this problem (**Figure 1C**). In this method, the first step involves coating the gel channel with fibrin gel containing a high density of EC (10 million cells/ml). This is done by injecting the gel and cell mixture and quickly aspirating it. The mixture of EC and normal human lung fibroblasts (NHLFs) of lower cell density is immediately injected in the gel. Subsequently, the device is flipped every 30 s for a few times to distribute the cells more evenly in the vertical direction. This method results in the middle region of the gel having a smaller diameter vessel network and perfusable openings at the sides. It has been demonstrated that CTC clusters consisting of a few cells are more easily trapped in the microvascular network (MVN) generated with two-step seeding.

One of the most attractive features of this method is the microvasculature architecture, which is similar to *in vivo* capillaries in terms of having complex branches and anastomoses, although the lumen diameter is usually still larger than capillaries. Perfusability is also one of the desired parameters to be evaluated when characterizing the MVN. This feature makes it a suitable tool for studying tumor extravasation. However, there is also a drawback: the vasculature architecture cannot be precisely controlled, which may, for example, make image analysis and fluid dynamics simulations more challenging.

Moreover, vasculogenesis usually requires 4–5 days for the vasculature to develop into a perfusable vasculature. This method is relatively time-consuming compared to the EC lining method, which generally only takes 1 day for the seeded ECs to form a confluent monolayer on the patterned lumen. Despite the longer time needed for establishment, the microvasculature network can be maintained for up to a few weeks, making it compatible with experiments requiring a longer time frame. To date, almost all works on vasculogenesis require early passage (up to P7) primary cells, which is more costly and may have batch-to-batch variation. Recent exploration of the use of hTert-immortalized human umbilical vein endothelial cells (HUVECs) and normal human lung fibroblasts (NHLFs) shows that immortalized HUVECs can form a perfusable vasculature network in the presence of Thy1+ immortalized NHLF as a substitute for low-passage primary NHLF (21).

Fibrinogen is a glycoprotein highly abundant in plasma. Thrombin cleavage of fibrinogen into fibrin exposes the interaction site that allows fibrin monomers to self-polymerize and form fibrillar structure (22). Fibrin gel is the most commonly used ECM material for forming microvasculature networks-on-a-chip *via* vasculogenesis. The final concentrations for fibrinogen and thrombin typically range from 2 to 3 mg/ml and from 0.5 to 2.0 U/ml, respectively. Other ECM components, such as collagen I or the mixture of fibrin and collagen I, have also been tested. However, the vessel network formed is not as complex and connected as that formed in fibrin gel only. Moreover, the incorporation of collagen I leads to gel contraction and detachment from the microfluidic channel (23). Perhaps pretreatment of the channel with adhesive such as glutaraldehyde might help resolve this issue. The following questions remain: Can fibrin be used as a representative ECM in the TME, which is well known for its collagen ECM? Can the relatively soft fibrin gel simulate the relatively stiff tumor ECM? Recently, there have been some reports on the application of other hydrogels on vasculogenesis, such as PEG-based hydrogel (24) and agarose supplementation to collagen or Matrigel (25). The hydrogels are able to support vessel network formation but further characterization of the vessel (e.g., perfusability, junctional integrity, and barrier function) is warranted.

#### 2.2.2 Angiogenesis

Angiogenesis is the formation of new vessels from an existing vessel under angiogenic stimulation. The two main mechanisms of angiogenesis *in vivo* are sprouting angiogenesis and intussusception or vessel splitting (26). Vascularizing tumor-on-a-chip using an angiogenesis method usually refers to sprouting angiogenesis as the vessel needs to grow into and reach the previously non-perfused region. This is more challenging to achieve with intussusception, which mainly gives rise to a new vessel in the area with some existing vessel.

The first step in this method is to create an endothelial monolayer through the EC lining method, by lining either the whole channel or only the side of the gel. Alternatively, the vessel can also sprout from an adjacent vascular bed formed by vasculogenesis (27). Under the stimulation of interstitial flow or certain growth factor gradients, such as the VEGF gradient (**Figure 1D**), which can be introduced by supplementing exogenous growth factors or coculture with stromal cells or certain types of cancer cells, ECs are activated and start to proliferate and sprout vessels toward the direction of the stimulus (28, 29). The direction of interstitial flow can also greatly affect angiogenic sprouting where interstitial flow in the direction opposite to vessel sprouting enhances angiogenesis while flow in the same direction suppresses vessel sprouting (27). However, it should be noted that angiogenic sprouts can sometimes be difficult to perfuse if the sprouts do not anastomose.

### 2.3 Heterotypic spheroid to improve intratumoral vascularization

Recently, quite a few groups have proposed introducing fibroblast and/or ECs to the tumor spheroid to improve vascularization, especially in the internal part of the tumor (17, 30–32).

To better vascularize tumor spheroid, Ahn et al. (17) use a heterotypic spheroid that comprises HepG2 liver cancer cells and blood ECs. In the presence of intratumoral vessels, the tumor is found to have higher expression of genes associated with aggressive behavior in cancer, such as EMT, cell migration, cell proliferation, and vessel development. To further vascularize the tumor, blood and lymphatic ECs are embedded together with the heterotypic spheroid in fibrin gel in the microfluidic channel. Interstitial flow is established to promote vessel network formation.

Besides forming spheroids from tumor cells and ECs, co-culturing cancer cells and fibroblasts can also improve vascularization. Wan et al. (30) tried two co-culture methods (1): mixing cancer cells and fibroblasts prior to seeding and (2) sequentially layering tumor spheroids with fibroblasts. They found that the latter method significantly improves vascularization in the internal part and the vicinity of the tumor (**Figure 1E**).

Park et al. (31) demonstrate the use of tri-culture spheroid comprising tumor cells, ECs, and fibroblasts. The tri-culture spheroid has a more robust intratumoral vasculature and connects better with the external microvasculature network. They observed the sprouting of ECs from the spheroid and the anastomosis with the external vessel. The improved vascularization results in higher tumor growth and more efficient delivery of drugs to the tumor core.

To form perfusable vessel lumen connected to the interior of tumor, Nashimoto et al. (32) tri-cultured HUVECs, fibroblasts, and MCF-7 as a spheroid in 96-well ULA, which was then embedded in collagen-fibronectin hydrogel at the middle channel of a three-channel microfluidic device. The sides of the lateral channels were lined with HUVECs. Both HUVECs from the sides and in the spheroid sprouted and anastomosed, forming vessels capable of perfusing the interior of the spheroid.

## 3 Heterotypic cellular interactions in the vascularized tumor microenvironment

### 3.1 Tumor–vessel interactions

Tumor-on-a-chip technology has recapitulated various phenomena of tumor–vessel interaction such as tumor angiogenesis, vessel destruction in pancreatic cancer, and mosaic vessel, where the vessel is composed of both ECs and cancer cells. Furthermore, it has also been used to study the importance of organ-specific EC.

**Figure 2:**
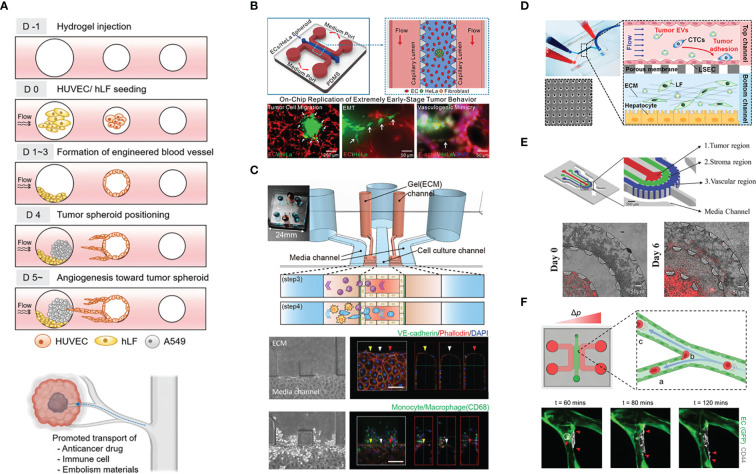
Example of the application of vascularized tumor-on-a-chip for mechanistic studies. **(A)** The study of tumor vascularization by angiogenesis and the promotion of therapeutic transport. Adapted with permission. Copyright 2022 **(D)** Kim et al. Advanced Healthcare Materials published by Wiley‐VCH GmbH. **(B)** The recapitulation of extremely early-stage tumor dynamics and interaction with blood vessel. Adapted with permission. Copyright 2021, American Chemical Society. **(C)** Elucidating the role of monocyte/macrophage in forming pre-metastatic niche. Adapted with permission. Copyright 2019 **(H)** Kim et al. Published by WILEY‐VCH Verlag GmbH & Co. KGaA, Weinheim. **(D)** Investigating the effect of tumor-derived extracellular vesicles in priming the pre-metastatic niche. Adapted with permission. Copyright 2020, American Chemical Society. **(E)** The concentric three-layer channels to study breast cancer cell migration and intravasation. Adapted with permission. Copyright 2018 WILEY‐VCH Verlag GmbH & Co. KGaA, Weinheim. **(F)** Investigation of tumor extravasation mechanism in a microvascular network enabled by the good live imaging resolution. Adapted under the terms of the Creative Commons Attribution License (CC BY). Copyright 2021 Offeddu et al.

Kim et al. (33) designed an alternative vascularized tumor-on-a-chip model where ECs and fibroblasts are seeded in two adjacent prepatterned channels. Mimicking the high interstitial fluid pressure of tumors, interstitial flow is introduced in a direction that can aid the transfer of fibroblast-secreted molecules toward the EC channel, which promotes angiogenic sprouting toward the fibroblast channel. It is interesting that the device allows spheroid introduction to the fibroblast channel after the main vasculature has matured, as early introduction of tumor cells might potentially have an adverse effect on vessel formation (34). In this study, they showed that tumor vascularization enhances therapeutic delivery, whether drugs, CAR T cells, or nanoparticles (**Figure 2A**).

Miller et al. (35) cocultured patient-derived renal carcinoma cells with HUVECs lining the hollow lumen created with rods. Under perfusion, the blood vessel structure was well preserved over time near the inlet while the vessel regressed near the outlet. In contrast, in the coculture, more prominent angiogenic sprouts were observed near the outlet. They hypothesized that the tumor secreted pro-angiogenic that concentrate near the outlet. The results of computational fluid dynamics (CFD) simulation also support their hypthesis.

A recent work by Nguyen et al. (36) investigated the interaction between a biomimetic pancreatic cancer duct and a blood vessel positioned 500 µm apart. Intriguingly, pancreatic ductal adenocarcinoma (PDAC) cells collectively migrated toward blood vessels and induced contact-dependent EC apoptosis. This phenomenon was mediated by activin and the receptor ALK7 expressed by PDAC cells. Their observation of this cancer hallmark was validated in a mouse ectopic tumor model and genetically engineered mouse models (GEMMs). Their results provide an explanation for the mechanism underlying PDAC hypovascularization despite high vascular invasiveness and explain the high CTC load in PDAC patients. Another study using MMTV-PyMT mouse tumor organoids (37) also described the formation of mosaic vessels as one of results of the interaction. Cancer cells were observed detaching from the fused organoids to the vascular lumen. In addition, the tumor organoid constricted or pulled the vessel.

Different cancer subtypes may have different interactions with the vasculature. As demonstrated by Gadde et al. (10), inflammatory breast cancer (IBC) cells did not disrupt ECs through anoikis as severely as non-IBC cells. Instead, IBC cells induced more angiogenic sprouting. VEGF expression levels were also found to be higher in IBC cells. During the course of the 16-day experiment, tumor clusters were found in the sprouted vessel. In terms of matrix degradation, the IBC microenvironment had increased porosity, supposedly due to degradation.

Usually, numerous spheroids or single cells are seeded in the ECM material to mimic cancer. However, in early-stage cancer, the tumor burden might not be that high. Thus, Li et al. (38) only added a single, small spheroid (30, 60, or 90 µm) into the ECM, together with ECs and fibroblasts, which formed a network through vasculogenesis; the tumor exhibited increased migration and spreading when ECs were present. They also observed chains of migrating cells and vasculogenic mimicry, which refers to the ability of cancer cells to organize themselves into vessel-like structures (39). (**Figure 2B**).

The importance of using the biologically relevant EC source is also demonstrated in the work by Gerigk et al. (40) Cultured in their glioblastoma (GBM) microfluidic-based model, ECs derived from the brain are shown to have lower permeability compared to HUVEC and lung HMVEC (human microvascular ECs) as a result of higher ZO-1 expression. Interestingly, the GBM cells migrate further when co-cultured with brain EC.

### 3.2 Interactions with immune cells

In addition to vasculature formation, the other significant advantage of the tumor-on-a-chip model is the incorporation of immune cells. This feature of immune cell incorporation can provide insights that are more relevant to the phenomena happening in the human body. The presence of vessel can better mimic the *in vivo* conditions during immune cell extravasation. Vascularized tumor-on-a-chip platforms have also been utilized to study immune cell polarization and exhaustion in the hostile TME. Finally, it has also been used to study the role of immune cells in cancer extravasation.

STING (STimulator of INterferon Genes) expression and cytokine secretion by tumors can induce T-cell chemotaxis. Using a vasculogenic approach, Campisi et al. (41) cocultured KRAS/LKB1 mutant lung cancer spheroids with HUVECs and observed a synergistic effect on cytokine production. Strikingly, the re-expression of LKB1 (upstream negative regulator of STING) in tumors did not show a significant increase in cytokine production in the coculture, suggesting a greater contribution from ECs. Apparently, the paracrine effect of cGAMP from tumor spheroids regulates STING expression in ECs. Consequently, vessel permeability increased, and ECs upregulate adhesion molecules for T-cell adhesion. Thus this work demonstrated an indirect mechanism of immune escape via vasculature priming.

Mollica et al. (42) investigated T-cell infiltration into the pancreatic cancer TME comprising Panc-1 cells, pancreatic stellate cells, and HUVECs, each seeded in different microfluidic channels. The model mimicked the increased vessel permeability in the presence of T cells. There was also increased infiltration of activated (through CD3/CD28 stimulation) T cells toward the PDAC compartment. Cytokine analysis revealed that the T cells in coculture of pancreatic cancer cells and stellate cells exhibited stronger activation, as indicated by higher IL-2 and IFNγ levels measured by a multiplex immunoassay. Upregulation of granzyme B, perforin, and Fas suggested that activated T cells were more cytotoxic than nonactivated T cells in the presence of HUVECs and Panc-1 cells. The tri-culture group showed lower levels of these genes, suggesting that pancreatic stellate cells diminished the inflammatory response and supported tumor growth.

By stacking tumor cell-seeded collagen gel with a porous membrane for culturing ECs in a microfluidic channel, Lee et al. (43) observed reduced T-cell transendothelial migration (TEM) and extravasation in the presence of tumor cells. ECs downregulated ICAM-1 and E-selectin due to tumor VEGF production, while the chemokines secreted from tumor cells still attracted T cells.

Humayun et al. (44) designed a microfluidic device to have a vessel channel that is close to one side of the gel to mimic the characteristic oxygen and nutrient tension in a large, necrotic tumor. The device was able to show a viability gradient across the gel. When used for NK cell TEM study, most of the extravasated cells migrated only up to 200 µm from the vessel. Thus, NK cells needed to be homogeneously resuspended in the gel for subsequent NK cell exhaustion experiments. Interestingly, they observed a lower NK cell proliferation rate and less responsiveness to chemokines on the distant side. Immune checkpoint inhibitor treatment alleviated exhaustion, but cytotoxicity was only partially restored at the distal side. In their previous work using a similar method (9), they observed poor antibody penetration into tumor spheroids, in part due to endocytosis.

Recently, the recapitulation of indirect signaling of tumor and T cells was confirmed *in vitro* (45). The researchers observed that the microvascular network cocultured with HepG2 cells (hepatocellular carcinoma cells) exhibited higher expression levels of FasL, which then induced the apoptosis of T cells. In vessel monoculture, hypoxia alone also induced the upregulation of FasL in ECs. Moreover, the MVN perfused with conditioned medium from HepG2 cells grown under hypoxic conditions also showed increased FasL expression. Subsequently, they interfered with this pathway by anti-Fas, anti-FasL, or pan-Caspase inhibitors and observed a significant reduction in the percentage of apoptotic T cells.

Kim et al. (46) used a basement membrane and EC-lined microfluidic channel flanked by collagen I gel on the lateral sides to study the role of monocytes/macrophages in establishing the premetastatic niche. Strikingly, monocytes increased vessel permeability by disrupting EC junctions and secreted MMP9 to disrupt the vascular barrier, promoting tumor extravasation. As monocytes/macrophages migrated in the lateral ECM, they left behind microtracks, which were then utilized by the tumor cells to more easily invade the ECM (**Figure 2C**).

Macrophage polarization toward the M2 (anti-inflammatory) phenotype has been implicated in promoting cancer progression. By culturing the microvascular network and tumor cells—in fibrin and Matrigel growth factor reduced (GFR) basement membrane matrix, respectively—in different microfluidic compartments, the work by Bi et al. (47) reproduced the angiogenic potential of the cancer, by comparing CD31 staining from the parental tumor. They also showed how the introduction of different macrophage lineages (nonpolarized, M1, and M2) can differentially affect the three parameters mentioned above. Moreover, they demonstrated how the device could be used for antibody-based drug studies. Finally, the uniform manifold approximation and projection (UMAP) from scRNA-seq revealed two EC populations, and immunostaining confirmed the different locations of the populations in the microfluidic device—the population related to tumor development and progression was located at the center of the vessel compartment, while the population related to the immune pathway and cell functions was located at the periphery of the vessel compartment and beyond. In addition, the introduction of M2 macrophages significantly increased the population related to the immune pathway and cell functions.

With their signature microvascular network, Boussommier-Calleja et al. (48) investigated the influence of monocytes on cancer extravasation. First, to elucidate whether TEM is necessary for monocytes to mature into macrophages, they compared CD68 (macrophage marker) expression between monocytes that exited to the stroma during perfusion and monocytes that were directly embedded in the gel with ECs and fibroblasts. They found that TEM was not necessary for monocyte maturation. Interestingly, they found that the same group of tumor cells in circulation differed in extravasation fate, suggesting that the inherent difference between cells, not merely their position, contribute to extravasation potential. The presence of circulating monocytes reduced the tumor extravasation rate, while the presence of macrophages in the stroma caused different changes in the extravasation rate in two different cell lines.

Using a microvasculature network-on-a-chip, Chen et al. (49) explored the role of inflamed neutrophils in tumor extravasation. In the inflamed state, endothelial ICAM-1 and neutrophil CD11b were upregulated to better support neutrophil and tumor cell aggregation. This heterotypic clustering prevented tumor cell loss due to fluid flow. In addition, the presence of an inflamed neutrophil cluster produced IL-8, and tumor cells produced CXCL-1. This chemotactic gradient caused neutrophils to undergo confined migration. In addition, IL-8 caused endothelial activation and barrier function disruption. Furthermore, the microfluidic device design, which included two large media reservoirs on top, allowed a steadier flow to be generated passively to continuously perfuse the microvascular network with neutrophils and tumor cells.

In another study on the role of neutrophils in tumor extravasation, an MVN chip model was used to complement mouse *in vivo* observations. The MVN model revealed that neutrophils increased tumor extravasation in a paracrine manner, facilitating cancer cell protrusion formation in the early phase of TEM (50).

In a microvasculature network-based extravasation assay, the introduction of platelets and neutrophils increased the tumor extravasation rate (51). Treatment with eptifibatide, an integrin β3 antagonist, not only prevented platelet aggregation but also reduced tumor PAI-1 and MMP-9 expression and interfered with tumor-EC adhesion. By blocking EC integrin β3, FAK and Src activation were reduced, which subsequently decreased VE-Cad phosphorylation and internalization, thus restoring the junctional barrier. Interestingly, the researchers retrieved and sorted the cells to perform Western blot analysis, but they did not mention the number of devices each sample was pooled from.

### 3.3 Interactions with other components

Thrombocytosis, the condition where blood platelet is elevated, is associated with adverse prognosis in various types of cancer (52–55). Platelets can contribute to tumor progression through a few mechanisms. In the circulation, platelet interaction with CTCs and ECs promotes tumor extravasation. In addition, platelet can directly affect primary or metastatic tumor, and this requires platelet extravasation to the tumor site (56). In microfluidic device, the platelet–tumor interaction has been recreated for both intravascular and extravascular context.

Using their OvCa-Chip device, which has vertically stacked microfluidic channels with the vessel in the lower compartment and ovarian cancer cells in the upper compartment separated by a porous membrane, Saha et al. (57) confirmed the role of tumor cytokines in activating the Src/ERK/FAK pathway, which led to VE-Cad and β-catenin downregulation in ECs, compromised EC junction integrity, and increased platelet extravasation. Statin, a class of cholesterol-lowering drug, can be repurposed to improve clinical outcome in cancer patients (58), although the exact mechanism is not fully understood yet. In the context of blood vessel, statin treatment has been shown to preserve endothelium junctional integrity (59, 60) and to have antiangiogenic effect (58). In this study, atorvastatin treatment “rescued” vessel integrity and reduced the number of extravasated platelets. Their findings were validated with patient biopsy samples.

An upgrade from their previous OvCa-Chip, the new OTME-Chip (61) has an additional gel channel on each lateral side of the tumor compartment separated by a micropillar, which allows the investigation of matrix invasion. Interestingly, the tumor only migrated to the ECM when extravasated platelets were bound to it. The interaction involved tumor galectin-3, which binds to platelet GPVI—a glycoprotein upregulated by shear stress. As tumor platelet interactions have been reported to promote chemoresistance, they utilized this platform to study the effect of cisplatin only versus cisplatin and antiplatelet drug combinations. Compared to cisplatin monotherapy, dual therapy showed reduced tumor invasion and proliferation. RNA-seq was performed, with the different cell types sorted prior to sequencing, revealing the upregulated pathways that can potentially be targeted, such as those regulating cell cycle.

Cancer cell-derived extracellular vesicles carry cargos such as oncoproteins and miRNA that may aid tumor progression by shaping the TME and establishing pre-metastatic niche (62). Recently, Kim et al. (63) investigated the effect of breast cancer cell-derived extracellular vesicles (cancer EVs) on priming in the liver premetastatic niche (**Figure 2D**). Utilizing two-layered microfluidics primed by cancer EVs, ECs underwent endothelial-to-mesenchymal transition (EMT), as shown by decreased ZO-1 intensity and increased vimentin and FAPa (fibroblast activating protein) intensity. Furthermore, priming enabled more cancer cells to adhere to ECs.

Mechanical cues such as stiffness is well known to affect cell function and behavior. Moreover, it has also been shown that cells can have mechanical memory; i.e., cell behavior can be regulated by its past mechanical environment (64, 65). Azadi et al. (66) showed that breast cancer cells previously cultured on stiffer substrates were associated with higher extravasation rates and migration distances in malignant breast cancer models. This trend was found to be correlated with increased MMP9 expression levels.

## 4 Unveiling the mechanisms of metastatic cascade using the vascularized tumor microenvironment

### 4.1 Invasion and intravasation

Nagaraju et al. (67) created a microfluidic model with concentric layers comprising, from inner to outer sides, breast cancer cells, collagen gel, and ECs that form a microvasculature network over a few days in culture. The incorporation of ECs enhanced the outward migration of invasive cancer cells, likely in a paracrine manner, as suggested by cytokine profiling. The presence of invasive cancer cells decreased vessel diameter and increased vessel permeability through VEGF secretion (**Figure 2E**).

Using the same microfluidic device setup, Truong et al. (68) cultured glioma stem cells (GSCs) and ECs to study invasion and extravasation. On-chip vasculogenesis requires serum, but serum exposure can induce GSC differentiation, so the authors first formed the microvascular network for 3 days and subsequently introduced the GSCs into the chip. The tumor cell chain migration was observed in both the GSC-on-a-chip model and their animal model. In the presence of vasculature, phosphorylated CXCR4 staining in GSCs showed a punctate pattern, suggesting its activation under the stimulation of CXCL12 secreted by ECs. When treated with AMD3100, a CXCR4 inhibitor, GSCs migrated over a shorter distance.

Using the vessel formed by lining ECs on rod-patterned channels, Wong & Searson (69) observed how the location of breast cancer cells with regard to the vessel wall and the mitosis of cancer cells can affect the intravasation rate. The rounding of tumor cells was proposed to exert mechanical stress on the EC junction, causing junctional adhesion to fail when cancer cells transmigrated successfully. Moreover, the fluid flow in the luminal side helped to detach the cancer cells into circulation.

### 4.2 Extravasation

Integrin signaling plays important roles in cell migration (70). Using an *in vitro* microvascular network, Gilardi et al. (71) investigated the Cdk5/Talin-1/FAK pathway in cancer cell TEM (transendothelial migration). Cdk5 controlled the phosphorylation of Talin-1, which regulates FAK phosphorylation. Silencing Talin-1 was associated with compromised vascular adhesion, while inhibiting FAK phosphorylation at S732 rendered the cells unable to perform TEM. The lower rate of extravasation observed *in vitro* was validated using an *in vivo* model.

Another study highlights the importance of tumor integrin β1 in extravasation (72). The depletion of integrin β1 did not affect TEM but arrested the tumor cell in the compartment between ECs and the basement membrane (BM) due to the impaired integrin β1-mediated interaction with laminin in the BM, which is crucial for the formation of actin-rich protrusions that breach the BM. In a mouse model, integrin β1 knockdown reduced metastatic colony formation, corresponding with the *in vitro* experimental conclusions.

In transformed breast ECs, activation of the EMT (epithelial-to-mesenchymal transition) program upregulated the expression of podocalyxin, which facilitated ezrin-mediated cortical actin polarization and initial TEM (73). The use of the MVN chip here allowed the imaging and quantification of tumor extravasation.

Extravasation requires cell−cell contact. Traditionally, the glycocalyx is seen as a protective barrier to prevent adhesion due to its dense and charged molecular nature (74). However, it can also be a ligand for cellular receptors (75). Using the MVN-on-a-chip model (**Figure 2F**), it was revealed that tumor cells shed hyaluronic acid, which accumulates on ECs, thus priming their adhesion with tumor cell CD44 and promoting extravasation (76).

Cells circulating in the vasculature is subjected to mechanical forces arising from fluid flow that can affect cellular response (77, 78). *In vivo*, the fluid flow is not limited to luminal flow (fluid flow in the vessel lumen). There are also transendothelial and interstitial flows, which are the flow of fluid entering or exiting the vessel and the flow of fluid in the matrix, respectively. A recent study examined the individual roles of transendothelial and luminal flow in tumor extravasation (79). Luminal flow was found to increase the tumor extravasation rate, while transendothelial flow accelerated the transendothelial migration process as well as migration in the surrounding matrix.

### 4.3 Other metastasis models

In ovarian cancer, tumor cells can shed into the peritoneal fluid and adhere to and invade the mesothelium to form peritoneal metastasis, which is common (80). To mimic the peritoneal metastatic site, the MVN-on-a-chip model can be modified to have an adipocyte coculture in the gel with mesothelial cells layered on one end. Then, ovarian cancer cells can be introduced from the reservoir on that end, mimicking the peritoneal cavity (81). In this tri-culture platform consisting of ECs, adipocytes, and mesothelium, vascular and mesothelial permeability were in a range similar to that observed *in vivo*. Using modular combination, it was observed that the mesothelium acted as a protective barrier, while the presence of ECs and adipocytes increased tumor attachment to the mesothelium. When cancer cell density was high, they clustered and invaded the mesothelium, while low-density cancer cells attached the mesothelium but were not capable of invading it, suggesting the importance of cancer cell density. Adipocytes are thought to support metastatic tumor growth by providing lipid as an energy source (82). Interestingly, lipid droplets were also present in cancer cells that had successfully invaded in the device.

## 5 Therapeutic screening

### 5.1 Chemotherapeutics

To study both drug-sensitive and drug-resistant clones of tumor cell lines in the same device, Wang et al. (83) designed two-layer vertical microfluidic channels, with the bottom layer lined with ECs and separated from the top by membrane. The top layer consisted of two narrower channels for the two tumor clones. This platform enabled simultaneous assessment of both clones while keeping them in different compartments. However, the interpretation of the results should be done with caution as another study has shown that the paracrine effect from one cell type (senescent MCF10A due to centrosome amplification) can affect the behavior of the other cell type (MDA-MB-468 breast cancer cells) (84). This is especially important for senescent cells, which is common in cancer drug treatment (85).

Jing et al. (86) demonstrated the use of a vertically stacked microfluidic device to study metastasis and drug sensitivity in MDA-MB-231 and HepG2 cell lines. Interestingly, the tumor clusters migrated along the flow direction of “blood vessel” fluid, and different mechanisms of TEM were observed—paracellular in MDA-MB-231 cells and transcellular in HepG2 cells. They also performed a tumor adhesion assay and drug sensitivity test for 5-fluorouracil (5-FU). There was a more pronounced decrease in the cancer progression parameters in vascularized tumor-on-a-chip compared to coculture in transwell inserts, suggesting the importance of ECs and fluid flow in modulating 5-FU efficacy. A similar setup was also used to evaluate the anti-tumor potency of partially acetylated chitosan oligosaccharide (87).

An open microfluidic device with a 96-well plate format was used to coculture the MVN and CRC cell lines (34). They found that primary endothelial progenitor cell (EPC) and HUVEC source and passage number affected their ability to form MVNs in their rhomboidal gel chamber. EPCs or HUVECs from all sources were able to form a network by day 6, but only ECs from three out of seven sources tested had a perfusable network. They also observed different network formation dynamics for different cell sources. Among the cell sources tested, one of the two HUVEC lines showed the most consistent network formation dynamics in the presence and absence of HCT 116 colorectal cancer cells (which might be important for drug screening control). The system was used to assess drug safety by assessing tumor growth, vessel length, and cell viability. Although some cell sources resulted in nonperfusable MVN, the chemotherapeutic 5-FU still reached the tumor through diffusion despite the drop in efficiency indicated by higher IC_50_ compared to the cell lines capable of forming a perfusable network. Thus, vessel network perfusability should also be considered when assessing drug safety and sensitivity.

By reproducing GBM cells in vertically stacked microfluidic channels, Lin et al. (88) identified that the presence of ECs maintained the stem-cell like nature of GBM cells and weakened the response to DNA alkylating agents, as shown by reduced 7-mG and 6-O-mG levels assessed *via* LC−MS.

### 5.2 Antiangiogenic therapy

To better vascularize tumor spheroids, Ahn et al. (17) used heterotypic spheroids comprising HepG2 liver cancer cells and blood ECs. They demonstrated that in the presence of intratumoral vessels, the tumor cells exhibited more aggressive metastasis. To further vascularize the tumor, blood and lymphatic ECs were embedded together with the heterotypic spheroid in fibrin gel in the microfluidic channel. Interstitial flow was established to promote vessel network formation. Then, the researchers tested the tumor response to axitinib, an antiangiogenic drug. Outside of the chip, the heterotypic spheroids showed a dose-dependent response to axitinib treatment. When vascularized on-chip, the tumor shows no significant dose-dependent decrease in area in response to treatment at the earlier time point, when the vessel network was not properly developed yet. However, the vasculature still regresses in a dose-dependent manner. When drug treatment is started by the time the vasculature has already been well established (day 5 postseeding on-chip), the tumor responded to axitinib treatment, suggesting the importance of intratumoral vascularization for drug delivery.

### 5.3 Targeted therapy

Hassell et al. (7) modeled lung cancer in an orthotropic manner where a low density of non-small cell lung cancer (NSCLC) cells was mixed with normal alveolar ECs and added into the top chamber and the EC-lined bottom chamber. Interestingly, NSCLC cells in the coculture exhibited a 12-day dormancy period, and proliferation resumed at a lower rate than that in the monoculture. In the presence of cyclic stretch mimicking breathing motion, the tumor exhibited higher resistance to tyrosine kinase inhibitors (TKIs). Additionally, EGFR expression and phosphorylation levels were decreased under cyclic stretch. This observation might explain the reduced growth rate of tumors under breathing motion. The IL-8 and VEGF levels were increased in the effluent flow from the coculture, while the IL-6 level was decreased.

### 5.4 Combination therapy

**Figure 3:**
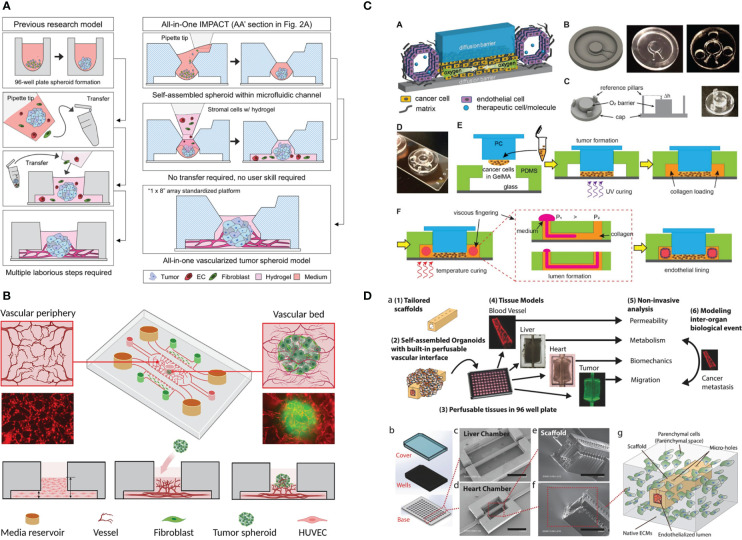
Example of the application of vascularized tumor-on-a-chip for therapeutics screening. **(A)** The all-in-one IMPACT device allows spheroid formation and vascularization on the same device, making it more user-friendly. Adapted with permission. Copyright 2022 Wiley Periodicals LLC. **(B)** The device design with a small hole on top of the device enabled spheroid introduction after microvascular network formation. Adapted with permission. Copyright 2022, American Chemical Society. **(C)** Device design allowing oxygen gradient generation with the gas-impermeable PC cap. Endothelial cells are seeded on lumen created by viscous finger patterning. Adapted under the terms of the Creative Commons Attribution License (CC BY). Copyright 2021 Ando et al. **(D)** The multi-organ InVADE platform that enables drug sensitivity and other organs’ toxicity test in one chip. Adapted with permission. Copyright 2017 WILEY‐VCH Verlag GmbH & Co. KGaA, Weinheim.

Kim et al. (18) recently developed an all-in-one IMPACT device, where spheroid formation and vascularization can be performed in the same device (**Figure 3A**). By injecting a small volume of cell suspension, the droplet was maintained by surface tension. After 24 h of spheroid formation, stromal cells and hydrogel were injected into the same space to vascularize the spheroid. The device was subsequently used to evaluate the effect of Taxol (chemotherapeutic) and Avastin (antiangiogenic drug) and the combination towards patient-derived cancer spheroid and vessel growth. After drug treatment from day 3 to day 5, all drug-treated groups experienced significant decrease in vessel area. Interestingly, Taxol monotherapy resulted in the highest decrease in tumor size change. Furthermore, the bottom of the device was detached, and the hydrogel was sectioned for high-resolution imaging. This was done to obtain clearer confocal images of vascularized mono- or heterotypic spheroids (cancer cells, fibroblasts, and ECs).

The vasculogenic-formed vasculature network mimics physiological size and architecture. However, in the commonly used microfluidic device, tumor cells can only be seeded together with ECs to form MVNs within tumors and are thus the size of tumor clusters cannot be finely controlled. In the work by Hu et al. (89), a small hole was punched on the central channel to introduce tumor spheroids after MVN formation (**Figure 3B**). However, the higher position of the spheroid in this design might potentially reduce the imaging quality. The platform was utilized to study the effect of HIF1a stabilization by inhibiting prolyl hydroxylase (PHD) on improving traditional chemotherapeutic outcomes. PHD inhibitor treatment reduced the EC apoptosis rate and vessel permeability (vessel normalization), leading to the more effective delivery of chemotherapeutics to the tumor.

### 5.5 Nanoparticles

Nanoparticles has recently emerged as a potential approach to treat cancer that can be engineered to have advantageous effect such as targeted-delivery and optimized drug release pattern (90), vascularized tumors-on-chips have been utilized to study how the presence of the endothelial barrier affects the delivery of micelle-based nanoparticles. As expected, the delivery rate of nanoparticles was the highest in 2D culture, followed by 3D spheroids and vascularized tumor-on-a-chip models (91). Separately, Wang et al. (92) investigated the effect of different mophologies of Gd2O3 NPs, namely sphere, scroll, and oblate, towards the tumor-killing efficacy. Scroll and oblate shape NPs had similarly good adhesion on the ECs but the oblate shape NPs was superior in supressing the growth of lung tumor due to the larger NP surface area (92).

### 5.6 Cell therapy

In addition to forming spheroids from tumor cells and ECs, sequentially layering tumor spheroids with fibroblasts can also improve vascularization within the tumor and its surrounding areas (30). Experiments with CAR T cells showed higher CAR T-cell recruitment, IFNg production, and apoptotic cell number in the tumor formed with fibroblast layering.

To perform drug screening under hypoxia, a steep oxygen gradient was created with a polydimethylsiloxane (PDMS) device and polycarbonate (PC) cap (11). Tumor cells were embedded in GelMA in the chamber underneath the PC cap, while the vessel was created by viscous fingering of the hollow space surrounding the tumor chamber (**Figure 3C**). Strikingly, no angiogenic response was observed, but the ECs directly invaded the tumor chamber by day 7. The vessel was found to be leaky as assessed by dextran perfusion. Most of the CAR T cells that flowed to the vascular channel did not attach, but when they did attach, they migrated against the flow, suggesting active migration.

### 5.7 Vascularized multi organs-on-a-chip

Recently, tumor-on-a-chip has been coupled with cells derived from other organs, such as the liver (8, 93) and heart (93, 94), to assess toxicity to other vital organs. This investigation can be achieved by coupling of various organ cell types in different compartments, such as growing cancer cells in one lateral channel and cardiomyocytes in the other lateral channel. The InVADE platform (**Figure 3D**) enables this arrangement by separating the tumor cells and organ-derived cells into different but interconnected 96-well plates. Alternatively, Ozkan et al. (8) created a PDMS chip with two lumenized hydrogel compartments, one seeded with tumor cells and the other had liver cells in it. ECs were seeded on the lumen and the two compartments were connected with tubing.

## 6 Discussion and conclusions

The introduction of vasculature into the tumor-on-a-chip method has allowed more accurate reproduction of the TME. It is highly important for the study of tumor–vessel interactions as well as being the platform for therapeutic evaluation, as drugs and immune cells circulate through the vasculature. The modular nature of microfluidic models makes them a robust tool to delineate the contribution of each factor and to study their interactions. In addition, compared to animal models, microfluidic models offer better control and reproducibility while simultaneously being rapid and cost-effective.

Perfusable vessels were successfully generated by coculturing ECs and stromal cells. However, there are limited examples of *in vitro* models where pericytes are present. Pericyte crosstalk with ECs is important for vessel maturation and endothelial barrier function (95, 96). HUVECs are the primary cell lines most commonly used in vasculature-on-a-chip formation. Nevertheless, it is known that vessel characteristics differ from organ to organ. For example, the liver has sinusoid capillaries, the kidney has fenestrated capillaries, and the brain has dense capillaries. For the blood−brain-barrier (BBB) in particular, a complex vessel model where pericytes and astrocytes are cultured together with ECs has been generated (13). ECs form the MVN, and pericytes and astrocytes obtain perivascular localization. To date, most of the studies on vascularized tumor-on-a-chip only incorporate blood vessels. However, in an *in vivo* context, lymphatic vessels are also an important route for metastasis in various types of cancer (97). Thus, lymphatic vessels have currently been introduced together with blood vessels for a tumor-on-a-chip model (17). These factors might lead to discrepant or even contradictory findings when compared to *in vivo* results.

Currently, microfluidic devices for organs-on-a-chip are usually PDMS-based with coverslip bottoms. PDMS is gas-permeable, nontoxic, and transparent, making it suitable for cell culture and imaging applications. However, the manufacturing of this device requires personnel skilled in microfabrication and special instruments that are not commonly found in biomedical laboratories. Furthermore, the fabrication process is somewhat time-consuming, and there is also concern about the nature of PDMS, which can absorb small-molecule drugs (98), making it not the most ideal material for use in drug screening. To enable a wider adoption of vascularized tumors-on-a-chip and organs-on-a-chip methods in general, a more standardized microfluidic device is needed. It is also preferred, especially in drug screening applications, that the device format is compatible with high-throughput systems. Currently, devices suitable for vascularized tumors-on-a-chip can also be made from plastic materials and have been made commercially available in various designs. Devices such as OrganoPlate^®^ Graft (Mimetas) (https://www.mimetas.com/en/organoplate-sup-sup-product-overview/ last accessed 2023-01-10) and OrganiX (AIM Biotech) (https://aimbiotech.com/ last accessed 2023-01-10) enable the grafting of single tumor spheroids or organoids on/in a vascular bed. For more general applications, devices such as IdenTX (AIM Biotech) are a more versatile option.

Another hurdle is that the workflow of cell isolation for downstream analysis is tedious and has a higher chance of contamination. Usually, the whole gel slab needs to be extracted from the device, and the ECM is digested. Subsequently, the cells are collected and sorted for further analysis. Although the small volume allows more replicates to be produced, it prevents the ability to perform conventional analyses, such as Western blotting, due to an insufficient cell number. It is still possible to perform them, but cells need to be pooled from several devices.

Tumor-on-a-chip analysis relies heavily on imaging tools and is currently incompatible with many conventional *in situ* assays. For example, in the 3D culture of cells embedded in gel, traction force microscopy analysis remains challenging and computationally expensive, and the potential alternative for quantifying cellular forces is fluorescence resonance energy transfer (FRET) tension sensors (99). To study calcium release in vascular smooth muscle cells surrounding the MVN, Cuenca et al. (100) transduced the cells with an ultrasensitive calcium sensor, GCaMP6f. Similar molecular tools can be exploited for other molecules of interest. Atomic force microscopy, a common tool to investigate tissue or cell stiffness, is also not compatible with the geometry of most microfluidic devices. Alternatively, Brillouin has recently been proposed to potentially solve this problem (101, 102). However, the instrument is not very common yet.

In conclusion, the development of *in vitro* vascularized tumor models has significantly advanced our ability to recreate a more biologically realistic cancer microenvironment in the laboratory. This has provided researchers with a more precise and accurate way to study the mechanisms of cancer and evaluate therapeutic response. With further refinement, standardization, and simplification of the technology, we are optimistic that *in vitro* microvasculature will gain wider adoption as a replacement for animal experimentation and play a crucial role in advancing personalized cancer therapy.

## Author contributions

CBXH wrote and reviewed the manuscript under supervision by T-YT. All authors contributed to the article and approved the submitted version.
